# Vestibular Function Measured Using the Video Head Impulse Test in Congenital Nystagmus and Vertigo: A Case Report

**DOI:** 10.3389/fneur.2021.690402

**Published:** 2021-06-08

**Authors:** Antonio Denia-Lafuente, Belén Lombardero

**Affiliations:** Unidad de Oído y Vértigo, Hospital Nuestra Señora del Rosario, Madrid, Spain

**Keywords:** congenital nystagmus, vertigo, vHIT, vestibular function, case report

## Abstract

In patients with congenital nystagmus (CN), the study of vestibular function is complicated by many factors related to the measurement of the vestibulo-ocular reflex (VOR) by means of caloric testing and the video head impulse test (vHIT), and to date no such studies have successfully employed the vHIT to evaluate vestibular function in these patients. We present a case with CN and vertigo in which peripheral vestibular function was evaluated using the vHIT system, including head impulse testing and the suppression head impulse protocol. We show that it is possible (a) to identify lateral VOR changes such as abnormalities resembling those produced by bilateral vestibular lesions, though not necessarily related to the same mechanism; (b) to identify peripheral VOR lesions of the vertical semicircular canals (SCC); and (c) to document compensation and recovery subsequent to these peripheral lesions during follow-up of patients with CN. vHIT is a useful tool that should be used to study vestibular function in patients with CN and vertigo, which could constitute a new clinical application of this technique.

## Introduction

The technical innovations behind the head impulse test (HIT) (1) and HIT-based applications to evaluate vestibular function with the aid of the search coil (2–4), video recording, and image-processing techniques, have led to the creation of a high-speed video-oculography system called video head impulse test (vHIT) (ICS Impulse device, Otometrics A/S, Taastrup, Denmark) (5, 6). Currently, the entire protocol (vHIT measurement system and HIT) is referred to as HIMP (head impulse testing), although both terms remain in use (7). The protocol includes a new variation called SHIMP (suppression head impulses) which, when used alongside the HIMP, completes the study of vestibular lesions (8). Ample evidence has shown the clinical utility of the vHIT when diagnosing unilateral and bilateral peripheral vestibular lesions (9–11) and to study the evolution of these lesions [spontaneous (12, 13) and after treatment (14–19)] to identify patients with poor prognosis for disability-preventing early rehabilitation (20, 21). vHIT can also be employed to monitor head impulse training and VOR ongoing compensatory strategies (12, 19, 22, 23), to study new clinical patterns evidenced on 3D video head impulse test (7, 24), and to diagnose central vestibular lesions, distinguishing these from peripheral lesions (25, 26).

Congenital nystagmus (CN) is an ocular motor disorder that can present at birth or in early childhood. It is mostly evident when patients attempt fixation, beating mainly in the horizontal plane. CN changes its shape in lateral gaze (often pendular, biphasic, or in the form of microsaccades) (27–29) and remains mostly horizontal in vertical gaze (28). Evidence from studies of peripheral vestibular function in patients with CN are scarce, and research using vHIT is lacking (30), mainly because calibration and quantification of eye movements is very problematic and sometimes impossible (31).

We report the peripheral vestibular function of a patient with CN and vertigo using the vHIT system. We have found no existing studies on the use of this technique to evaluate these patients.

## Case Presentation

### Case Description

A 73-year-old male retiree after a 45-year career as a television broadcasting technician with no previous difficulties with balance, vision, or hearing presented to our unit with complaints of acute vertigo and imbalance when getting out of bed over a number of days 2 months prior. These were the first such episodes in his life. His past history included acute myocardial infarction, type 1 diabetes, congenital nystagmus (CN), farsightedness, and astigmatism, though he was otherwise healthy and remained an active member of a string-instrument band. The patient denied having any symptoms affecting his hearing.

He reported to the emergency department during the acute phase due to an inability to stand, after which he continued to present instability and imbalance over 1 month, even with mild movements. His instability and imbalance were less intense on presentation to our unit, although his condition continued to prevent him from going out alone and from driving. The patient was examined by an ear, nose, and throat specialist (without specific diagnosis, treated with betahistine) and a neurologist. A magnetic resonance imaging scan of the brain and inner ear revealed no abnormalities. During his first visit, his dizziness handicap inventory (DHI) was 30.

### Diagnostic Tests and Follow-Up

**Figure 1:**
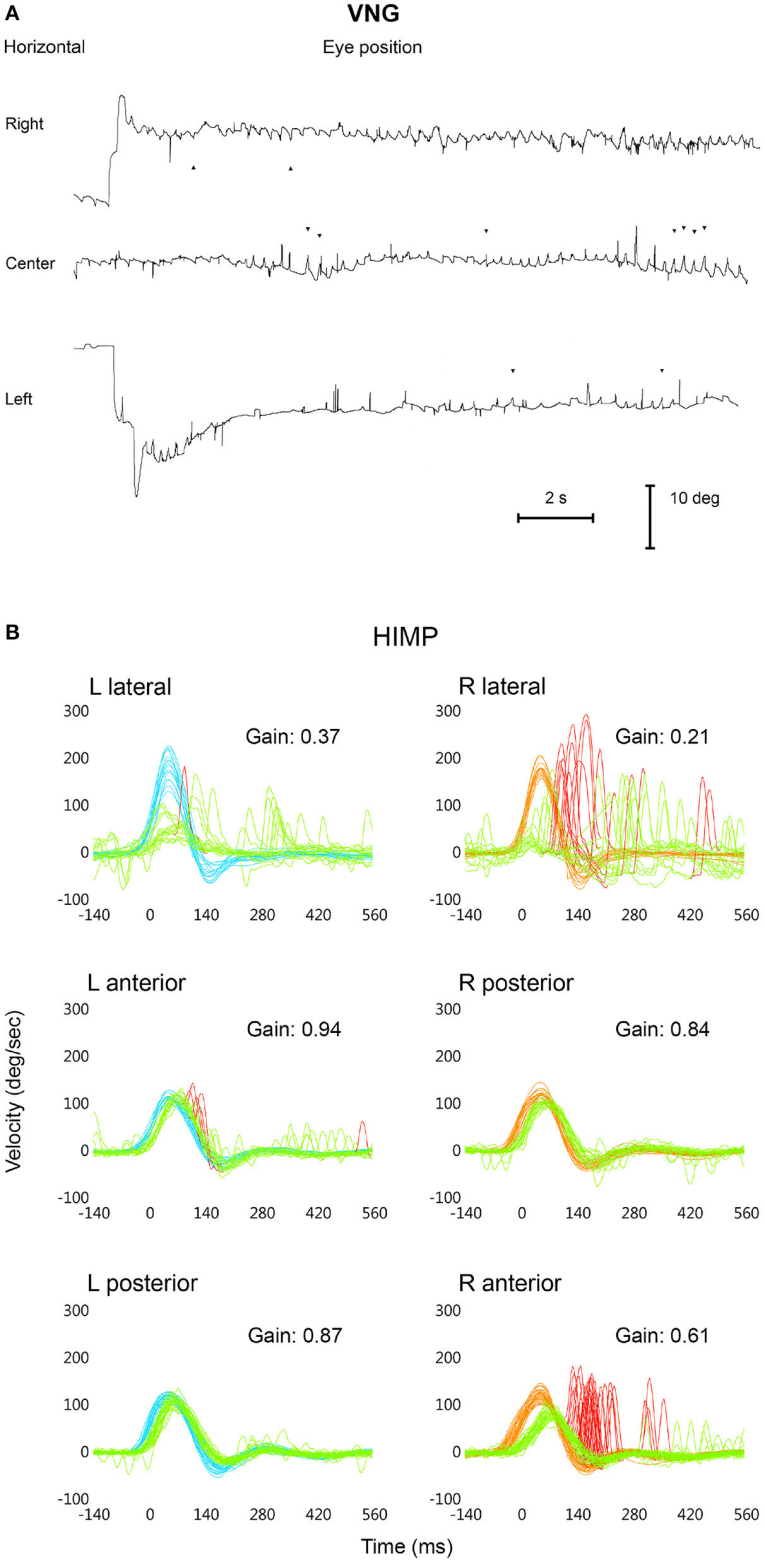
Horizontal videonystagmography (VNG) recording of the left eye showing the congenital nystagmus looking to the right (upward), to the center, and to the left (downward) **(A)**. vHIT superimposed head [right: red; left: blue] and eye [green] velocity records in degrees/second (*y* axis) vs. time in ms (*x* axis) during HIMP trials for each semicircular canal, including mean values for VOR gain; the “Spontaneous Nystagmus” check box was not selected **(B)**.

A clinical examination and videonystagmography (VNG) was performed to test his eye movement (Interacoustics VN415). With the patient looking at the center, a high-frequency horizontal nystagmus (3–4 beats/s) was observed, with no well-defined or even alternating fast phase initially and pseudopendular left beating afterwards; the nystagmus changed direction when the patient looked to the right, becoming more pendular. Gaze to the left showed a much less evident irregular pseudopendular left beating nystagmus (Figure 1A). The nystagmus prevented reliable assessment of vestibular function through a battery of clinical (spontaneous nystagmus, head shaking, vibration-induced nystagmus, HIT) and VNG tests. A Romberg test revealed increased static body sway without falling. His gait was wide-based, he was unsteady and had difficulty maintaining balance; these symptoms worsened with head movements.

The first HIMP protocol (Figure 1B) was performed without selecting the check box “Spontaneous Nystagmus” (SN not selected) (32). Calibration of eye movements took abnormally long, though measurement was possible (31). We experienced great difficulty in generating bilateral horizontal VOR and fast, high-velocity compensatory eye movements were recorded, with impulses mainly to the right [80% refixation saccades, 53% of which were overt, with a PR score of 52% (33)] and only some slower saccades to the left (6% covert) (Supplementary Video 1), with a mean VOR gain of 0.21 to the right and 0.37 to the left. Vertical VOR showed mean gain values of 0.61 for the right (R) anterior semicircular canal (SCC) [lower than normal age-range values (32)] with 100% of saccades identified as overt (PR score, 45%) (Supplementary Video 2). The mean VOR gain of the left (L) anterior SCC was within the normal range, with 31% covert saccades (PR score, 22%); mean VOR gain values for both posterior SCCs were within normal limits and no saccades were detected in these channels.

Cervical vestibular-evoked myogenic potentials (cVEMPs) were normal (Supplementary Figure 1). Computerized dynamic posturography (CDP) showed a vestibular pattern with visual dysfunction, average stability of 63%, hip-strategy movements, and center-of-gravity alignment displaced forward with falls in conditions 5 and 6 (Supplementary Figures 2A,B).

Pure tone audiometry evidenced bilateral sensorineural hearing loss (SNHL). The loss was mild at high frequencies in the right ear and mild to moderate in the middle-to-high frequency range for the left ear, compatible with presbycusis (Supplementary Figure 3).

On his first visit to our unit (2 months after onset of symptoms), the patient was initially diagnosed with acute vestibular syndrome (AVS), probably due to a right superior vestibular neuritis with incomplete compensation of the vestibular deficit.

The patient underwent a customized vestibular rehabilitation program in our unit and in his home, including exercises for gaze stabilization, VOR adaptation, habituation acquisition, and balance (34). VOR adaptation also included the following: passive and then active lateral head impulses to both sides with vHIT monitoring (12, 19) and effective pitch-up and pitch-down head impulses. The patient was also instructed to perform these exercises in the home and carry out a range of activities in different environments to recover activities of daily living.

**Figure 2:**
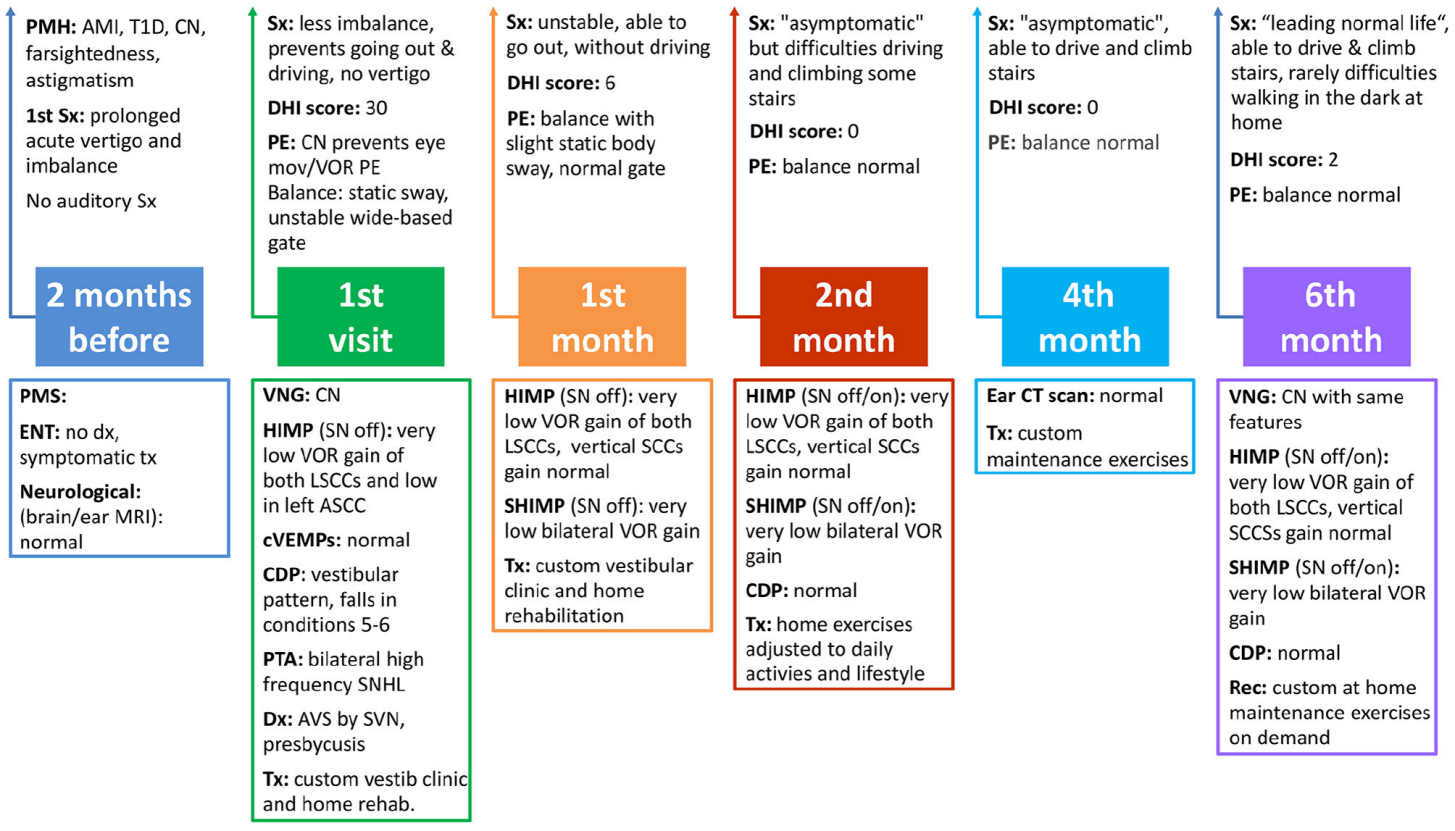
Timetable including the clinical information obtained during patient visits. PMH, past medical history; AMI, acute miocardial infarction; T1D, type 1 diabetes; CN, congenital nystagmus; Sx, symptoms; Dx, diagnosis; PMS, previous medical studies; N, normal; Tx, treatment; PE, physical examination; SN off, HIMP/SHIMP protocol performed without selecting the check box “Spontaneous nystagmus”; SN on, HIMP/SHIMP protocol performed selecting the check box “Spontaneous nystagmus”; LSCCs, lateral semicircular canals (SCCs); ASCC, anterior SCC; CDP, computerized dynamic posturography; Rec, recommendations.

As shown in Figure 2, the patient had follow-up visits at 1, 2, 4, and 6 months, including DHI and physical examination. HIMP and SHIMP tests were performed during all visits (except at month 4) in the same conditions as the 1st visit. In contrast, SN was selected at months 2 and 6, thus allowing comparison of the VOR in the two vHIT conditions. We performed a CDP at months 2 and 6, a temporal bone CT scan at month 4, and a VNG at month 6 of follow-up. Time series records for vHIT recording of all six canals vHIT recording timeseries are included as Supplememtary Material together with the impulse rejection/aceptance rate (Supplementary Figures 4–7).

**Figure 3:**
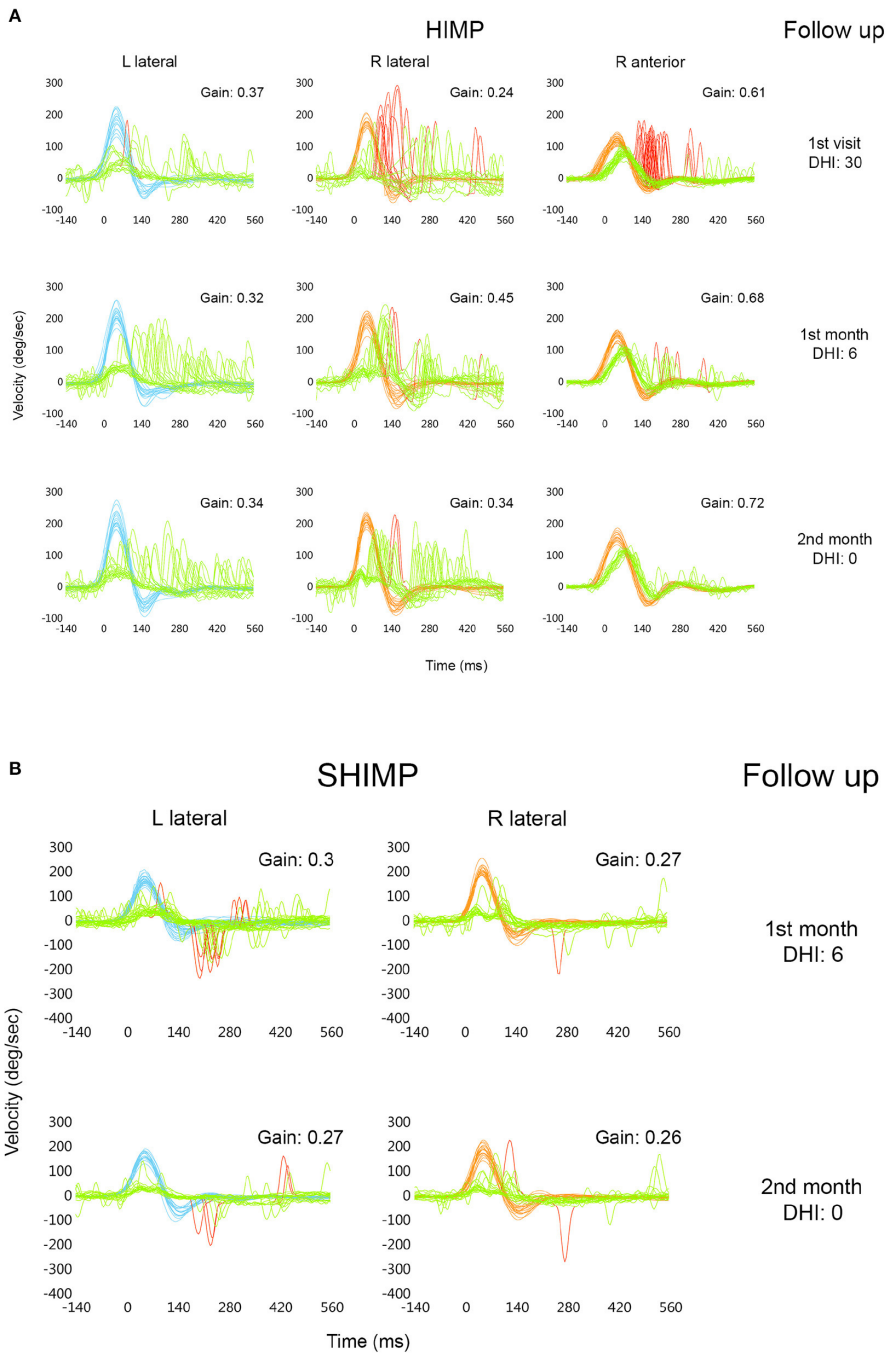
vHIT comparative superimposed head [right: red; left: blue] and eye [green] velocity in degrees/second (*y* axis) vs. time in ms (*x* axis), during HIMP trials of both lateral SCCs and of the R anterior SCC including VOR mean gain values, obtained at the 1st visit and at the 1st and 2nd month of follow-up with DHI scores (right column); “Spontaneous Nystagmus” check box not selected **(A)**. Similar vHIT comparative superimposed head and eye velocity in degrees/second (*y* axis) vs. time in ms (*x* axis) during SHIMP trials obtained at the 1st and 2nd month of follow-up with DHI scores (right column); “Spontaneous Nystagmus” check box not selected **(B)**.

The patient reported significant improvement at 1 month of follow-up, with less imbalance, though he continued to refrain from leaving the home and driving (DHI: 6). At month 2, he reported “leading a normal life” (DHI: 0), although he was still unable to drive comfortably and sometimes had difficulties climbing stairs. HIMP test results for both lateral SCCs performed at 1 and 2 months of follow-up and with calibration are shown in Figure 3A, together with those from the first visit (to allow for comparison along follow-up). We recorded mean VOR gain values for lateral impulses to the left of 0.32 at month 1 and 0.34 at month 2, and 0.45 and 0.34, respectively, for impulses to the right. Rapid saccadic compensatory eye movements were observed during both visits, showing similar unilateral patterns not identified as refixation saccades by vHIT with impulses to the left; with impulses to the right, only 10% of overt saccades were identified at the 1st month and 9% at the 2nd (80% on first visit). Mean VOR gain of the R anterior SCC at 1 month of follow-up improved from 0.61 (first visit) to 0.68 (normal age-range values), with 15% overt saccades (100% on the initial visit) (Figure 3A); at month 2, the mean gain was 0.72 with no presence of saccades (Figure 3A). At month 1 and 2, mean VOR gain in both posterior and in the L anterior SCCs were within the normal range for the patient's age, and no refixation saccades were found (data not shown in Figure 3A).

SHIMP testing protocols carried out at months 1 and 2 showed a mean VOR gain of 0.27 and 0.26 with impulses to the right and 0.3 and 0.27, respectively, to the left (Figure 3B), with a small number of anticompensatory saccades observed only at 1 month of follow-up, with impulses to the left.

**Figure 4:**
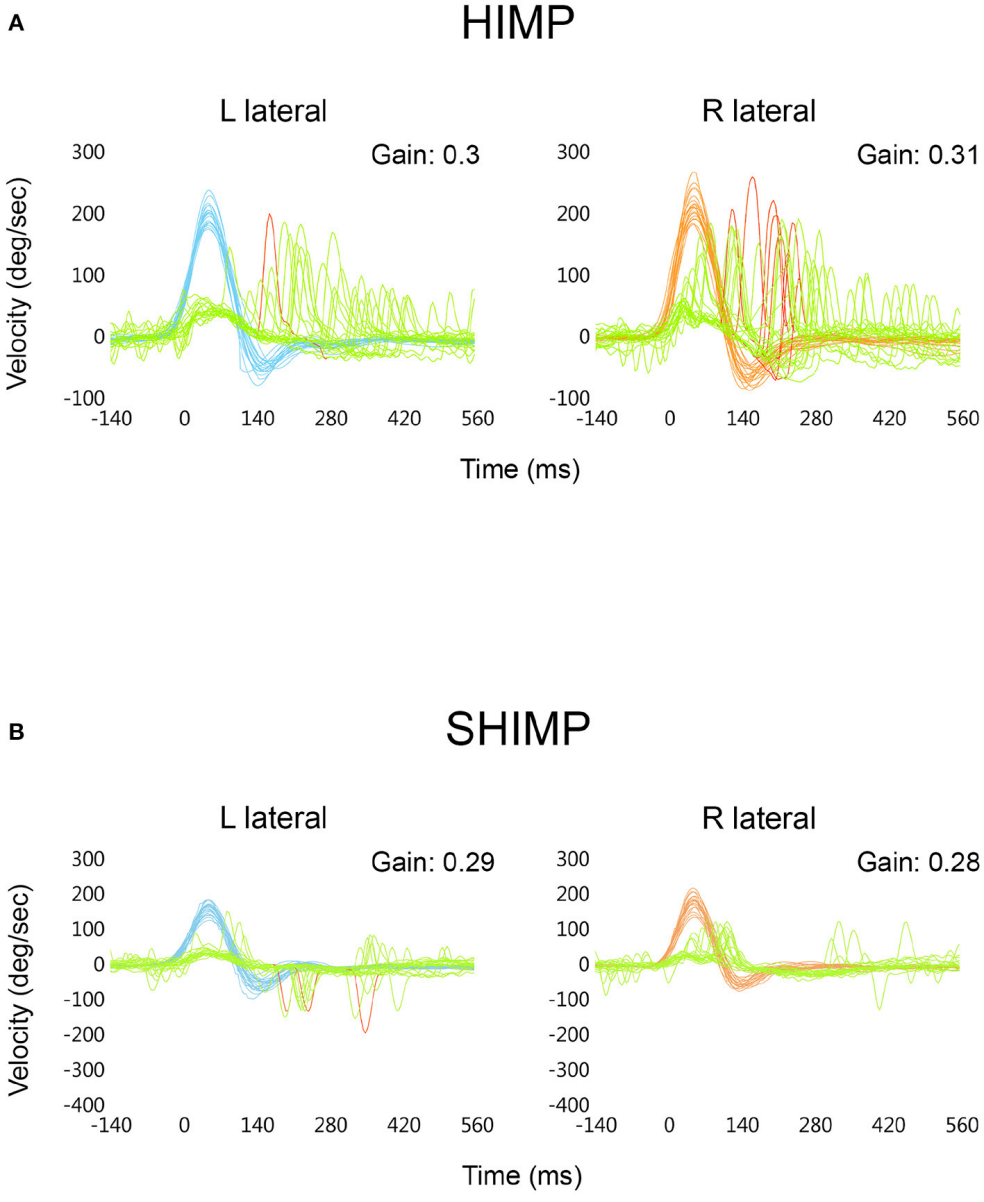
vHIT comparison of superimposed head [right: red; left: blue] and eye [green] velocity expressed in degrees/second (*y* axis) vs. time in ms (*x* axis) during HIMP trials of both lateral SCCs with the “Spontaneous Nystagmus” check box selected. Included are mean VOR gain values obtained at 2 months of follow-up **(A)**. Similar vHIT comparison of superimposed head and eye velocity in degrees/second (*y* axis) vs. time in ms (*x* axis) during SHIMP trials; “Spontaneous Nystagmus” check box selected. Included are mean VOR gain values obtained at 2 months of follow-up **(B)**.

At 2 months of follow-up, lateral HIMP trials (SN selected) (Figure 4A) showed similar mean VOR gain values (0.3 to the left and 0.31 to the right) as with SN not selected (Figure 3A), with 5% of refixation overt saccades identified to the left and 30% to the right. Each vertical SCC showed a mean VOR gain that was within the normal values for the patient's age (32) (Supplementary Table 1), without refixation saccades. SHIMP trials at 2 months of follow-up (Figure 4B) also revealed similar mean VOR gain values (0.29 to the left and 0.28 to the right) in both vHIT conditions (Figure 3B), without anticompensatory saccades.

CDP at 2 months was normal (average maximum stability, 82%).

At 4 months of follow-up the patient was asymptomatic and had recovered the ability to drive and climb stairs (DHI: 0). Balance and an ear CT scan were normal. Customized maintenance exercises were advised if needed (Figure 2).

At 6 months the patient was “leading a normal life with occasional slight difficulty walking in the dark at home” (DHI: 2). Balance and CDP were both normal. VNG showed CN with the same features as before. Lateral HIMP test results resembled those at month 2, with slightly higher HIMP-assessed mean VOR gain values with lateral impulses to the right in both vHIT conditions (Supplementary Table 1). Vertical HIMP trials also showed mean VOR gains that were within the normal age-based range (Supplementary Table 1). SHIMP mean VOR gain values were also similar to those at month 2 in both vHIT conditions (Supplementary Table 1), with some anticompensatory saccades upon impulses to the right (SN selected).

The patient adhered closely to the prescribed treatment and tolerated the treatments delivered at the clinic well. His self-report and subjective and objective outcome measures indicated appreciable improvement during follow-up (Figure 2).

## Discussion

One of our initial observations is that despite the difficulties in using the vHIT to calibrate and consequently quantify eye movements in patients with CN (31), calibration can be performed in some of these patients, although the process may take longer than usual, as in the patient reported here. In our experience (unpublished), calibration can also be performed as usual in some patients with CN and, in others for whom calibration is not possible, using a default calibration (32). Differences among patients could be related to the nystagmus type or waveform and foveating ability or the time required for foveation (29), among other factors.

Our report shows that in this particular patient with CN and vertigo, peripheral vestibular function can be assessed with the vHIT, providing information on the VOR of the six SCCs.

For the first time in a patient with CN and vertigo, vHIT evidenced a severe decrease in VOR gain in both lateral SCCs. This is a similar finding to our observations in patients with bilateral vestibular lesions (11), although the patient described here had no previous dizziness or oscillopsia, and his past history and clinical findings were unrelated to any known causes of such lesions (35), including thiamin deficiency (no alcoholism, ataxia, gaze nystagmus, diplopia, or gut surgery; normal neurological/MRI study). Persistence of very low lateral VOR gains with high-velocity bilateral saccade-like compensatory eye movements after symptoms and acute vHIT findings recovery could be related to a previous clinical form or variant of chronic bilateral vestibular abnormality of both lateral SCCs associated with the CN. We believe that CN characteristics (27–29) could interfere with other horizontal eye movements that also take place in a lighted area with the eyes open such as those of the lateral VOR evaluation carried out by vHIT (6). In fact, inappropriate retinal slip and loss of horizontal gaze stability not related to an abnormal VOR could be a mechanism that hinders the development of the horizontal VOR, giving rise to low horizontal VOR gain in some patients as has been previously shown (36). If so, this mechanism and the very low gain could have taken place at birth or somewhat later, although without leading to previous symptoms or being related to the recent episode of vertigo and posterior transient instability and imbalance. This absence of prior symptoms may be due to other adaptative mechanisms to improve gaze stability and dynamic visual acuity and to suppress oscillopsia and related symptoms as has been reported in the majority of patients with CN (12, 37). On the other hand, a very low previous lateral VOR gain prevents accurate assessment of the possible impact on this parameter and a rather disjointed or scattered saccade pattern (33, 38) in the R lateral SCC observed on the first visit cannot be related to the acute vertigo episode.

In our patient, vHIT also identified a decreased VOR gain of the R anterior SCC on the first visit with associated overt refixation saccades. These findings and their subsequent recovery (12), including clinical symptoms, are consistent with right acute vestibular loss, probably related to a right superior vestibular nerve lesion whose precise diagnosis cannot be completed as the oVEMPs were not available (right saccular and posterior SCC function not affected) (13, 39, 40).

Our results also show that CN does not interfere with vertical VOR function, which has made a valuable contribution to the diagnosis and follow-up of this patient and suggests that the function of vertical SCCs should be assessed in other cases with CN.

Despite the significant clinical improvement observed between months 1 and 2, the patient experienced persistent deficits as evidenced by his inability to drive comfortably and difficulty climbing stairs. These deficits disappeared, however, at month 4, probably due to complete compensation and recovery resulting from vestibular rehabilitation (20, 34). Taking into account the contribution of the visuo-oculomotor system to vestibular compensation-recovery (20, 41), we must highlight that CN did not interfere.

Interviews during follow-up illustrated that the most important outcomes for the patient included improvements in instability, imbalance, daily activities (going out, driving comfortably, climbing stairs, walking in the dark), and general lifestyle enjoyment. Overall, the patient stated that he was very pleased with the outcome of the interventions.

There were no technical limitations to the performance of the tests in this case as observed in other patients (31). Although the vHIT manual recommends selecting the check box “SN” when this condition is present (35), the tests were carried out with SN unselected as in the first visit, with results consistent with the established diagnosis and evolution. Comparing the HIMP and SHIMP protocols at month 2 and 6 carried out with different settings, the results were similar and the difference was clinically irrelevant. The limitations of this study are mainly related to the near total absence of both lateral VORs and the doubts raised as to their possible mechanisms and clinical interpretation. Are they due to a bilateral vestibular lesion? Are they a consequence of central adaptative mechanisms related to CN? We think that the absence of symptoms (outside of the acute vertigo episode) in this as in the majority of patients with CN (37, 42), together with previous evidence showing hyporreflexia of the LSCs in other patients with CN (36) (findings similar to those of our patient) are a consequence of these central adaptative mechanisms. In our opinion, the acute episode of vertigo including recovery took place based on the CN and its related mechanisms. As we mentioned, a utricular injury could not be investigated because oVEMPs were not available, which constitutes a limitation for the diagnosis. Consequently, and including the limitations inherent to single-patient studies, further evidence is required to precisely determine vestibular function in patients with CN, with and without vertigo.

In summary, although this is a single case, to our knowledge it is the first to open a new possibility of clinically studying patients with vestibular symptoms and CN using the vHIT. In our case, the association of CN with chronic very poor or almost absent lateral VOR with no symptoms other than those of an acute transient vestibular injury is a very interesting finding which should be studied in more patients to clarify its mechanisms. With what we know so far, we cannot rule out a relationship with central adaptation mechanisms associated with CN rather than with a bilateral vestibular injury. The possibility of routine study of vertical SCC function considerably expands the diagnostic and follow-up possibilities in these patients. Taking into account the difficulty of studying lateral SCCs, this study should be complemented by other tests.

In conclusion, our results could represent a first approach to establish evidence that broadens the clinical applications of vHIT in patients with dizziness/vertigo and CN.

## Data Availability Statement

The original contributions presented in the study are included in the article/Supplementary Material, further inquiries can be directed to the corresponding author/s.

## Ethics Statement

The studies involving human participants were reviewed and approved by CEIm: Hospital Universitario La Princesa, Madrid, Spain. The patients/participants provided their written informed consent to participate in this study.

## Author Contributions

AD-L performed the vHIT tests, collected the clinical data, and wrote the manuscript with the assistance of BL. BL developed and was in charge of the vestibular rehabilitation protocol.

## Conflict of Interest

The authors declare that the research was conducted in the absence of any commercial or financial relationships that could be construed as a potential conflict of interest.
